# Association of gut microbiota and gut metabolites and adverse outcomes in biliary atresia: A longitudinal prospective study

**DOI:** 10.1097/HC9.0000000000000550

**Published:** 2024-10-17

**Authors:** Vandana Jain, Matthew J. Dalby, Emma C. Alexander, Charlotte Burford, Holly Acford-Palmer, Iliana R. Serghiou, Nancy M.Y. Teng, Raymond Kiu, Konstantinos Gerasimidis, Konstantina Zafeiropoulou, Michael Logan, Anita Verma, Mark Davenport, Lindsay J. Hall, Anil Dhawan

**Affiliations:** 1 Paediatric Liver, GI and Nutrition Centre and Mowatlabs, King’s College Hospital, London, UK; 2 Quadram Institute Bioscience, Norwich Research Park, Norwich, UK; 3 Institute of Microbiology and Infection, College of Medical and Dental Sciences, University of Birmingham, Birmingham, UK; 4 Human Nutrition, School of Medicine, Dentistry and Nursing, College of Medical, Veterinary and Life Sciences, University of Glasgow, Glasgow Royal Infirmary, Glasgow, UK; 5 Department of Infection Science, Paediatric Liver, GI and Nutrition Centre and Mowatlabs, King’s College Hospital, London, UK; 6 Department of Paediatric Surgery, King’s College Hospital, London, UK; 7 Norwich Medical School, University of East Anglia, Norwich Research Park, Norwich, UK

**Keywords:** biliary atresia, liver transplantation, microbiota, short-chain fatty acid

## Abstract

**Background:**

The Kasai portoenterostomy (KPE) aims to re-establish bile flow in biliary atresia (BA); however, BA remains the commonest indication for liver transplantation in pediatrics. Gut microbiota-host interplay is increasingly associated with outcomes in chronic liver disease. This study characterized fecal microbiota and fatty acid metabolites in BA.

**Methods:**

Fecal samples were prospectively collected in newly diagnosed BA infants (n = 55) before and after KPE. Age-matched healthy control (n = 19) and cholestatic control (n = 21) fecal samples were collected. Fecal 16S rRNA gene amplicon sequencing for gut microbiota and gas chromatography for fecal fatty acids was performed.

**Results:**

Increased abundance of *Enterococcus* in pre-KPE BA and cholestatic control infants, compared to healthy infants, was demonstrated. At the early post-KPE time points, increased alpha diversity was revealed in BA versus healthy cohorts. A lower relative abundance of *Bifidobacterium* and increased *Enterococcus*, *Clostridium*, *Fusobacterium*, and *Pseudomonas* was seen in infants with BA. Fecal acetate was reduced, and fecal butyrate and propionate were elevated in early post-KPE BA infants. Higher post-KPE alpha diversity was associated with nonfavorable clinical outcomes (6-month jaundice and liver transplantation). A higher relative abundance of post-KPE *Streptococcus* and *Fusobacterium* and a lower relative abundance of *Dorea*, *Blautia*, and *Oscillospira* were associated with nonfavorable clinical outcomes. *Blautia* inversely correlated to liver disease severity, and *Bifidobacterium* inversely correlated to fibrosis biomarkers. *Bifidobacterium* abundance was significantly lower in infants experiencing cholangitis within 6 months after KPE.

**Conclusions:**

Increased diversity, enrichment of pathogenic, and depletion of beneficial microbiota early post-KPE are all factors associated with nonfavorable BA outcomes. Manipulation of gut microbiota in the early postsurgical period could provide therapeutic potential.

## INTRODUCTION

Biliary atresia (BA) is a disease characterized by fibro-obliteration of the biliary tree, presenting in infancy with obstructive jaundice that blocks the flow of bile from the liver to the gut.^1^ The etiology of BA remains uncertain but likely involves genetic and developmental factors that result in an immune response targeting the bile ducts. The Kasai portoenterostomy (KPE) surgery relieves the bile duct obstruction and re-establishes the flow of bile by creating a bilio-enteric conduit. However, jaundice clearance is only achieved in 60% of infants, and the progression of liver disease to cirrhosis that requires liver transplantation (LT) often continues even in infants with successful jaundice clearance.^2^ BA remains the commonest cause of LT in pediatrics. Post-KPE pharmacotherapy that aims to dampen fibro-inflammatory processes has not translated into improved native liver survival (NLS).

Over the last decade, the development of high-throughput genetic sequencing techniques has facilitated advanced detection and classification of the complex ecosystem of the *gut microbiota* (gut bacterial community) and their encoded genes, the *gut microbiome.* An emerging role for the gut microbiota in disease processes has been described, with gut microbiota perturbations (often referred to as dysbiosis) being described in numerous chronic disorders, including across the spectrum of liver disease in adult cohorts.^3^ The term “gut-liver axis” highlights the close anatomical and functional relationship between the liver and the gut microbiota. Metabolites produced by gut microbes, such as short-chain fatty acids (SCFAs) and secondary bile acids, are involved in modulating host metabolism^4^,^5^ and are being investigated in liver disease.

From as early as the 1980s, studies based on culturing of fecal samples have identified alterations in the gut microbiota in BA. Cholangitis, the inflammation of the biliary system, after KPE, provides the most convincing evidence of gut microbiota pathogenesis in BA, with potential pathways including the reflux of gut bacteria through the bilio-enteric conduit or bacterial translocation through the enterohepatic circulation.^6^ In light of data implicating gut microbiota pathogenesis in cholangitis, as well as in adult cholestatic and cirrhotic disease,^7^ sequencing techniques are identifying gut microbial disturbance in BA.^8^,^9^,^10^,^11^,^12^,^13^,^14^,^15^,^16^

In this prospective longitudinal study, a comprehensive analysis of the fecal microbiota and paired fecal fatty acid in BA pathogenesis and disease progression was conducted.

## METHODS

### Patient and control cohorts

In this prospective longitudinal single-center study, infants were recruited into 3 separate cohorts between January 2016 and December 2019: (i) BA, (ii) healthy control (HC), and (iii) cholestatic control (CC).

#### BA cohort

All infants with newly diagnosed BA, confirmed by intraoperative cholangiogram and/or liver biopsy pathology at King’s College Hospital (KCH), were candidates for the present study. Longitudinal assessment at baseline (pre-KPE), 6 weeks (6w), 12 weeks (12w), and 24 weeks (24w) post-KPE time points was performed for all infants. Exclusion criteria included previous bowel resection, candidate for primary LT, an extrahepatic disease associated with significant morbidity/mortality, and international candidates unable to attend longitudinal study visits (see Supplemental Methods for Clinical BA Protocol, http://links.lww.com/HC9/B57).

#### HC cohort

HC infants were recruited from the local community at 1 of the following chronological age-matched time points to the BA cohort: (i) 6 weeks (pre-KPE equivalent), (ii) 3 months (6w-post-KPE equivalent), (iii) 5 months (12w-post-KPE equivalent), and (iv) 8 months (24w-post-KPE equivalent). HC infants were not the same infants followed up longitudinally. Inclusion criteria included >37 weeks gestation and exclusively breastfed from birth. Exclusion criteria included any surgical intervention or postnatal medication administration.

#### Non-BA CC cohort

Infants presenting to KCH for investigation of conjugated jaundice (defined as conjugated fraction >20% total bilirubin) between the ages of 4 weeks and 2 months of age were recruited to the study as CCs. Exclusion criteria included previous bowel resection and subsequent BA diagnosis.

### Study data and clinical endpoints

#### All cohorts

Demographic, dietary, and pharmacotherapy characteristics were recorded for all 3 study cohorts (BA, HC, and CC) at baseline (pre-KPE time point and equivalent). The predominant baseline milk diet, reported by parent(s), from birth to initial feces collection, was summarized as (i) exclusively breast-fed, (ii) mixed breast/formula–fed, and (iii) formula-fed. The administration of high medium-chain triglyceride (MCT) milk before feces collection in BA/CC cohorts was also documented. For BA and CC infants, administration of ursodeoxycholic acid and/or postnatal antibiotics before initial feces collection was documented. Post-KPE and equivalent time points, demographic, dietary, and pharmacotherapy characteristics for BA and healthy infants were recorded. Dietary characteristics were documented using 3-day pre-visit dietary diaries, completed by parent(s): (i) exclusively breastfed, (ii) exclusively MCT-fed, (iii) mixed breast/MCT-fed, (iv) standard formula–fed, and (v) weaned versus non-weaned. “Weaned” refers to the process of introducing solid food.

#### BA cohort

Biochemical, hematological, and anthropometric data were collected at all time points (pre-KPE and 6w-, 12w-, and 24w-post-KPE). Biochemical parameters included total bilirubin (TB, µmol/L), AST (IU/L), GGT (IU/L), and albumin (g/L). Hematological parameters included international normalized ratio and platelet count (×10^9^/L). Liver stiffness measurement (kPa) was measured using transient elastography (Fibroscan) (see Supplemental Methods for transient elastography details, http://links.lww.com/HC9/B57). Mathematical scores, Pediatric End-Stage Liver Disease (PELD), and AST-to-platelet ratio index were calculated at each time point (see Supplemental Methods for scoring systems, http://links.lww.com/HC9/B57). Anthropometric parameters included weight (kg) and length (cm) measurements required for PELD calculation.

Primary clinical outcome measures were (i) 6-month jaundice clearance (BA-JC, TB <20 µmol/L by 6 mo after KPE: BA-J, TB ≥20 µmol/L by 6 mo after KPE) and (ii) 2-year NLS (BA-NLS, NLS by 2 y after KPE; BA-LT, LT/listed for LT by 2 y after KPE). Favorable clinical outcomes were defined as BA-JC and BA-NLS, and nonfavorable clinical outcomes were defined as BA-J and BA-LT. The secondary clinical outcome measure was cholangitis (see Supplemental Methods for cholangitis definitions, http://links.lww.com/HC9/B57).

### Fecal sample collection

Fecal samples were collected from all BA and HC infants at pre-KPE and post-KPE (6w-, 12w-, and 24w) equivalent time points. Fecal samples from CC infants were collected at the pre-KPE time point only. Samples were collected directly from the nappy using a sterile spatula and pot. All samples were kept refrigerated during transport at approximately +4°C for a maximum of 24 hours before being processed. Fecal samples were (i) stored at −80°C for DNA extraction and sequencing and (ii) mixed with an equal volume of 1 M sodium hydroxide (NaOH) and stored at −80°C, for fatty acid analysis.

### DNA extraction, 16S rRNA gene amplicon sequencing of fecal samples, and sequence analysis

FastDNA Spin Kit for Soil (MP) was used to extract genomic DNA from feces following the manufacturer’s instructions, with an extended 3-minute bead-beating. DNA concentration was quantified using a Qubit 2.0 fluorometer (Invitrogen) (see Supplemental Methods for 16S rRNA gene amplicon sequencing of fecal samples and sequence analysis, http://links.lww.com/HC9/B57).

### Determination of fecal SCFAs

SCFAs, acetate, propionate, and butyrate, were measured using gas chromatography (Agilent 7820A; Agilent Technologies) with a DB-WAX UI column on diethyl ether extracts, with nitrogen as the carrier gas.

### Statistical analysis

Continuous variables comprising non-normal data were reported as median values and IQR. Two independent groups were analyzed using the Mann-Whitney *U* test. Multiple independent groups were analyzed using the Kruskal-Wallis test, with pairwise comparisons performed using the Dunn (1964) procedure and Bonferroni correction. Categorical variables were reported as frequency and percentages (%). Pearson chi-square test was used to analyze categorical variables. The Fisher exact test was used to analyze categorical variables if expected cell frequencies are <5. A *p* value <0.05 was considered statistically significant for all tests. Mann-Whitney *U*, Kruskal-Wallis, and chi-square analyses were performed using IBM SPSS version 28.

#### Microbial diversity

Microbial diversity, at the genus level, was calculated using the vegan package version 2.6-4. The number of genera or species was determined for each sample, and the Shannon diversity index and Inverse Simpson diversity index were calculated accordingly.

#### Microbiota composition

The overall composition of the 16S rRNA gene sequence data was analyzed using NMDS (nonmetric multidimensional scaling). These plots were generated in R Studio using the vegan package version 2.6-4, employing a Bray-Curtis dissimilarity calculation. To determine the statistical significance of differences in microbial community structure based on infant metadata variables, permutational MANOVA was conducted using the Adonis function from the vegan R package version 2.6-4. Arrows and genus labels on the NMDS plots indicate bacterial genera driving the separation of points on the NMDS plots. To test for the differential abundance of genera and fecal fatty acids in relation to the infant cohort group and clinical outcome variables for the infant samples, the Maaslin2 package (Microbiome Multivariable Associations with Linear Models)^17^ was used. The analysis in Maaslin2 was performed using default options recommended for the software package, with an association being considered significant at a *p* value threshold of *p* < 0.05 and *q* value of <0.25 after correction for multiple testing using the Benjamini-Hochberg false discovery rate method. A genus-level association with a false discovery rate–adjusted *p* value <0.25 has commonly been considered statistically significant in previous microbiome studies. The Maaslin2 analysis was performed using Benjamini-Hochberg false discovery rate multiple testing correction, with a significance threshold of 0.25 used as the default threshold recommended for the Maaslin2 package. For microbiota genus abundance data, the analysis method “LM” was used with a minimum prevalence cutoff of 0.1, and an abundance cutoff of 0.1 was applied, with no normalization method used because the data input was previously normalized. For SCFA data, the analysis method “LM” was used with a minimum prevalence cutoff of 0 and an abundance cutoff or 0 applied, with the normalization method “TSS” and transformation method “LOGIT” applied to the data. Each infant cohort group and clinical outcome variable was tested individually as a fixed effect. For analyses involving more than 1 time point, the individual participant ID was included as a random effect.

#### Correlation analysis

Pearson’s correlation with correction for multiple genus testing (using the Benjamini-Hochberg method) was conducted to determine (a) associations between disease severity and relative abundance of the top 10 most genera and (b) associations between SCFA concentrations and the relative abundance of the top 10 genera. Variables were normalized using the bestNormalize package (version 1.9.1). Individual correlations were plotted using ggplot2 (version 3.1.4) in R as a scatter plot with a line of best fit determined by linear regression.

#### Ethical statement

The experimental protocol was approved by the London-Harrow Research Ethics Committee (REC 15/LO/1966) in accordance with the ethical standards established in the 1964 Declaration of Helsinki. Informed consent was obtained from the parent/guardian before specimen collection. CC samples were stored under the Biobank Ethical Agreement (REC 18/WA/0009), and access to samples was granted by the Biobank Access Committee.

## RESULTS

### Characteristics of pre-KPE fecal microbiota and SCFA profiles in BA, compared to age-matched control cohorts

Fifty-five infants with newly diagnosed BA were recruited before KPE, and followed at 6w-, 12w-, and 24w after KPE. In addition, 18, 19, 18, and 17 “separate” HC infants were recruited at equivalent ages to pre-KPE, 6w-, 12w-, and 24w-post-KPE time points. Twenty-one non-BA cholestatic infants were recruited at an equivalent age to the pre-KPE time point (see Supplemental Results for further details on CC, http://links.lww.com/HC9/B57). Fecal sample collection and 16S rRNA gene amplicon sequencing were successful in 33 BA, 17 HC, and 19 CC infants. Gas chromatography measurement of fecal fatty acids was successfully obtained from 34 BA, 11 HC, and 15 CC infants. Factors contributing to the missing DNA sequencing data included insufficient stool sample available or DNA quantification too low for sequencing. Factors contributing to missing fatty acid data included insufficient stool samples available or insufficient material for extraction after freeze-drying.

Table 1 shows baseline characteristics for the total infant cohort recruitment; Supplemental Tables S1 and S2, http://links.lww.com/HC9/B57, show baseline characteristics for fecal microbiota and fecal SCFA subcohorts, respectively. Demographic characteristics were similar between BA and HC infants at baseline, while there were fewer vaginal deliveries and lower birthweights among CC infants. Baseline diet was similar between BA and CC cohorts but significantly different between these “diseased” cohorts and the healthy cohort, with 33% of BA and 29% of CC infants exclusively breastfed compared to 100% of healthy infants. The majority of BA (82%) and CC (71%) infants were already receiving specialized high MCT formula milk. UCDA and postnatal antibiotic use was higher in CC versus BA infants. CC infants displayed a milder cholestatic phenotype (lower TB and GGT levels) compared to BA infants.

**TABLE 1:** Baseline characteristic comparison between pre-KPE BA and age-matched HC and CC cohorts for total cohort recruitment

	Total cohort recruitment					
Baseline characteristics	BA, n=55	HC, n=18	CC, n=21	BA vs. HC, *p*	BA vs. CC, *p*	CC vs. HC, *p*
Demographics						
Age at time point (d), median (IQR)	49 (17, 127)	40 (29, 61)	51 (22,95)	0.82	0.07	0.16
Mode of birth delivery (vaginal)	31 (56)	12 (67)	5 (24)	0.58	***0.02***	***0.01***
Sex (M)	33 (60)	12 (67)	13 (62)	0.78	0.1	0.1
Prematurity (<37 wk)	6 (11)	0 (0)	5 (24)	0.33	0.17	0.05
Birth weight (kg), median (IQR)	3.09 (2.75, 3.50)	3.31 (3.08, 3.63)	2.7 (1.79, 3.06)	0.09	***0.004***	***<0.001***
Laboratory parameters						
TB (µmol/L), median (IQR)	130 (102, 148)	—	95 (61, 131)	—	***0.008***	—
GGT (IU/L), median (IQR)	366 (242, 724)	—	165 (88, 429)	—	***0.009***	—
Pharmacotherapy						
UDCA	33 (60.0)	0 (0)	18 (86)	—	0.05	—
Postnatal antibiotics	19 (35)	0 (0)	17 (81)	—	***<0.001***	—
Predominant diet from birth						
Exclusively breastfed	18 (33)	18 (100)	6 (29)	***<0.001***	0.79	***<0.001***
Mixed breast/formula–fed	25 (46)	0 (0)	8 (38)	—	0.61	—
Formula-fed	12 (22)	0 (0)	7 (33)	—	0.38	—
MCT administration						
MCT-fed	45 (82)	0 (0)	15 (71)	—	0.36	—

*Note*: Data are n (%) unless stated otherwise. Statistical significance is represented as *p* < 0.05.

Bold and italic values are significant *p*-values < 0.05.

Abbreviations: BA, biliary atresia; CC, cholestatic control; HC, healthy control; KPE, Kasai portoenterostomy; MCT, medium-chain triglyceride; TB, total bilirubin; UDCA, ursodeoxycholic acid.

### Comparison of pre-KPE BA fecal microbiota and SCFA profiles against age-matched control cohorts

**FIGURE 1:**
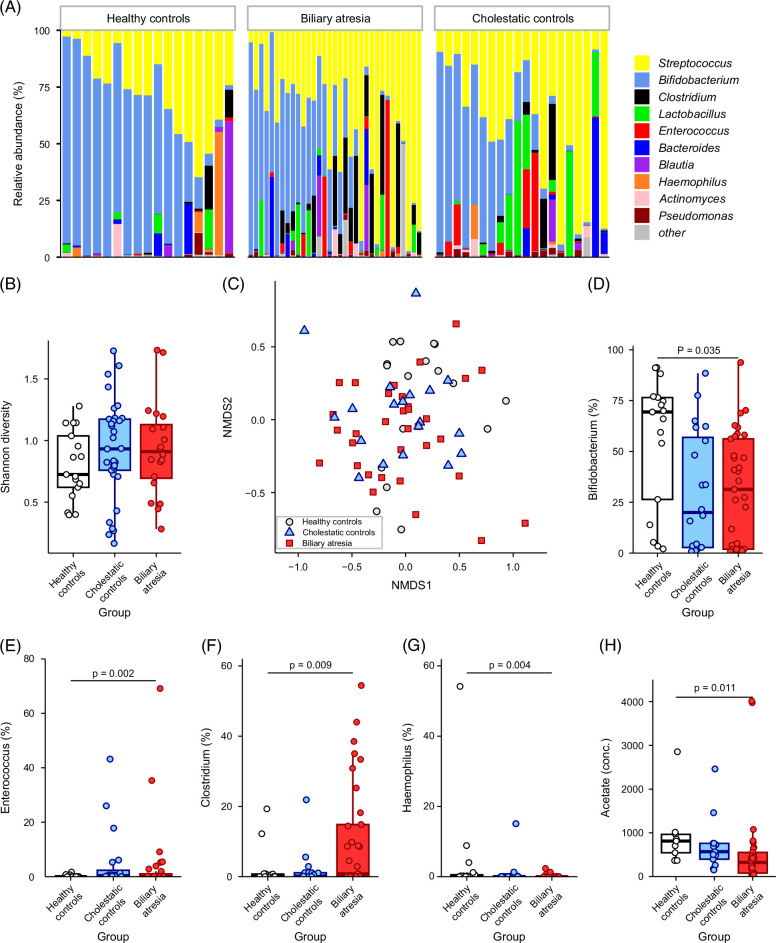
Pre-KPE composition of fecal microbiota and SCFAs in BA infants compared to healthy and cholestatic infants. (A) Pre-KPE relative abundance of the 10 most abundant bacterial genera in BA infants displayed as stacked bar plots compared to healthy and cholestatic control infants; (B) Shannon diversity of pre-KPE BA, age-matched healthy, and cholestatic infants. (C) NMDS plot showing clustering of genus level pre-KPE fecal microbiota (Stress = 0.18). PERMANOVA test: *R* ^2^ = 6.6%, *p* = 0.034. Pairwise analysis: HC versus BA, *p* = 0.066, HC versus CC: *p* = 0.091, BA versus CC: *p* = 1. Relative abundance of (D) *Bifidobacterium*, (E) *Enterococcus*, (F) *Clostridium*, and (G) *Haemophilus* between the infant groups. (H) Concentration of fecal acetate between infant groups. Statistical analysis of genus differences determined using Masslin2 with BA set as the reference group. Boxplots represent the median and interquartile range. Statistical significance was determined as *p* < 0.05. Abbreviations: BA, biliary atresia; CC, cholestatic control; HC, healthy control; KPE, Kasai portoenterostomy; NMDS, nonmetric multidimensional scaling; SCFA, short-chain fatty acids.

To examine the gut microbiota composition at the pre-KPE time point, the 10 bacterial genera with the highest abundance were visualized as stacked bar plots for BA, HC, and CC infants (Figure 1A). *Streptococcus* and *Bifidobacterium* were visually dominant in all 3 groups, with *Enterococcus* and *Clostridium* being more apparent in BA and CC infants. Microbial diversity was similar between the 3 groups (Figure 1B, Supplemental Figure S1A, http://links.lww.com/HC9/B57). Clustering samples using NMDS, based on a Bray-Curtis matrix, showed distinct clustering between BA and healthy infants (PERMANOVA test: *R* ^2^ = 6.6%, *p* = 0.034), with no separation between BA and CC infants (Figure 1C). The differential abundance of genera between groups was determined using the MaAsLin2 package with BA as the reference group. The relative abundance of *Bifidobacterium* (Figure 1D) was lower (*p* = 0.035) in BA infants than in healthy infants, while the relative abundance of *Enterococcus* (*p* = 0.002) and *Clostridium* (*p* = 0.009) was higher in BA (Figures 1E, F), compared to healthy infants, but similar to CC infants. *Haemophilus* was also lower in BA infants compared to HC infants (Figure 1G).

Fecal SCFA concentration analyzed using the MaAsLin2 package, with BA as the reference group, showed lower acetate (*p* = 0.011) in BA at pre-KPE compared to healthy infants (Figure 1H). Butyrate concentrations also tended to be higher (*p* = 0.056) in BA compared to healthy infants (Supplemental Figure S1B, http://links.lww.com/HC9/B57). Fecal SCFA concentrations were similar between BA and cholestatic infants.

### Longitudinal assessment of BA fecal microbiota

**FIGURE 2:**
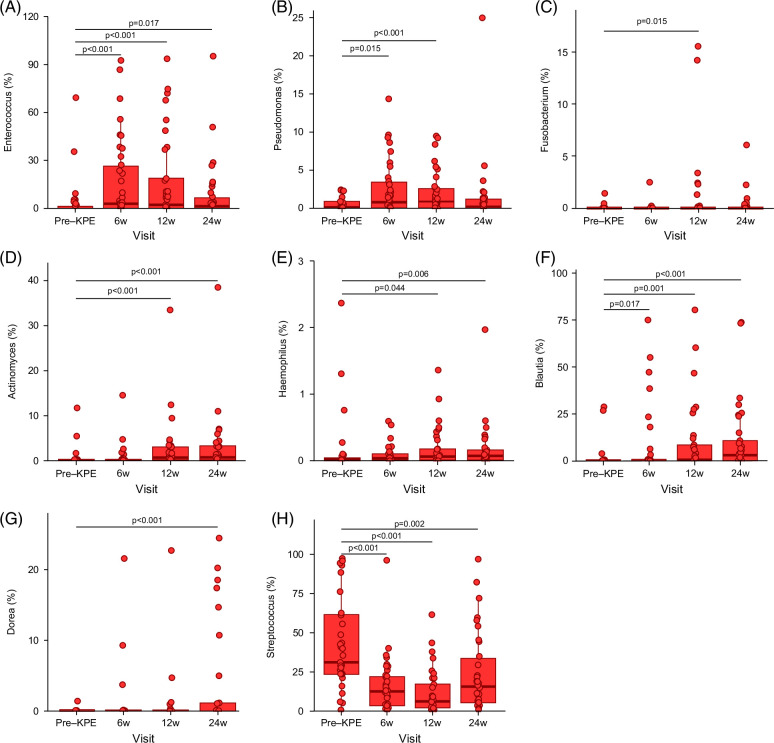
Longitudinal microbiota composition in BA infants. Longitudinal relative abundance of (A) *Enterococcus*, (B) *Pseudomonas*, (C) *Fusobacterium*, (D) *Actinomyces*, (E) *Haemophilus*, (F) *Blautia*, (G) *Dorea*, and (H) *Streptococcus* in BA infants across time points. Abbreviations: BA, biliary atresia; KPE, Kasai portoenterostomy.

Differential abundance analysis of BA microbiota, with pre-KPE as the reference group, was conducted to understand their progression over time. *Enterococcus*, *Pseudomonas*, *Fusobacterium*, *Actinomyces*, and *Haemophilus* abundance was higher after KPE compared to before KPE (Figures 2A–E). *Blautia* abundance progressively increased over time (Figure 2F), Dorea was higher at 24w after KPE (Figure 2G), and *Streptococcus* abundance was lower after KPE (Figure 2H).

### Comparison of post-KPE fecal microbiota and SCFA profiles against age-matched HC

Fecal microbiota and fecal SCFAs were compared longitudinally at 6w-, 12w-, and 24w-post-KPE in BA and age-matched healthy infants. Table 2 and Supplemental Table S3, http://links.lww.com/HC9/B57, compare characteristics between BA and HC at all time points for fecal microbiota and fecal SCFA subcohorts, respectively. Breastfeeding was significantly lower in BA infants at all time points; pharmacotherapy administration was abundant in BA, compared to pharmacotherapy-naïve healthy infants. Weaning rates were similar between both cohorts.

**TABLE 2:** Baseline characteristic comparison between longitudinal BA and age-matched HC and CC subcohorts for fecal microbiota subcohort

	Fecal microbiota analysis								
	6w-post-KPE			12w-post-KPE			24w-post-KPE		
Confounder variable	BA, n = 38	HC, n = 17	*p*	BA, n = 33	HC, n = 14	*p*	BA, n = 33	HC, n = 16	*p*
Baseline characteristics									
Age at time point (wk)	12.4 (10.7, 15)	13.3 (13, 13.9)	0.2	19.4 (17, 22)	21.3 (20, 23)	0.1	32.9 (30, 35)	34.2 (32.8, 35.4)	0.2
Mode of birth delivery (vaginal)	23 (61)	11 (65)	0.8	18 (55)	8 (57)	0.9	20 (61)	12 (75)	0.3
Sex (M)	22 (58)	7 (41)	0.3	18 (55)	7 (50)	0.7	17 (52)	8 (50)	0.9
Prematurity (<37 wk)	4 (11)	0 (0)	0.2	2 (6)	0 (0)	0.4	4 (12)	0 (0)	0.2
Birth weight (kg)	3.04 (2.7, 3.5)	3.4 (3, 3.5)	***0.04***	3.01 (2.8, 3.4)	3.4 (2.9, 3.6)	0.2	3 (2.7, 3.3)	3.4 (3, 3.6)	0.07
Pharmacotherapy									
UDCA	37 (97)	0 (0)	***<0.001***	32 (97)	0 (0)	***<0.001***	32 (97)	0 (0)	***<0.001***
Antibiotics between time points	38 (100)	0 (0)	***<0.001***	15 (46)	0 (0)	***0.002***	17 (52)	0 (0)	***<0.001***
Diet									
Exclusively breastfed	1 (3)	17 (100)	***<0.001***	1 (3)	14 (100)	***<0.001***	0 (0)	16 (100)	***<0.001***
Mixed breast/ MCT-fed	7 (18)	0 (0)	0.1	5 (15)	0 (0)	0.1	4 (12)	0 (0)	0.2
MCT-fed	30 (79)	0 (0)	***<0.001***	29 (88)	0 (0)	***<0.001***	24 (73)	0 (0)	***<0.001***
Extensively formula-fed	0 (0)	0 (0)	NA	3 (9)	0 (0)	0.2	7 (22)	0 (0)	0.05
Weaned	0 (0)	0 (0)	NA	6 (18)	2 (14)	0.7	29 (88)	16 (100)	0.2

*Note*: Data are n (%) unless stated otherwise. Statistical significance is represented as *p* < 0.05.

Abbreviations: BA, biliary atresia; CC, cholestatic control; HC, healthy control; KPE, Kasai portoenterostomy; MCT, medium-chain triglyceride; NA, assessment not appropriate; UDCA, ursodeoxycholic acid.

**FIGURE 3:**
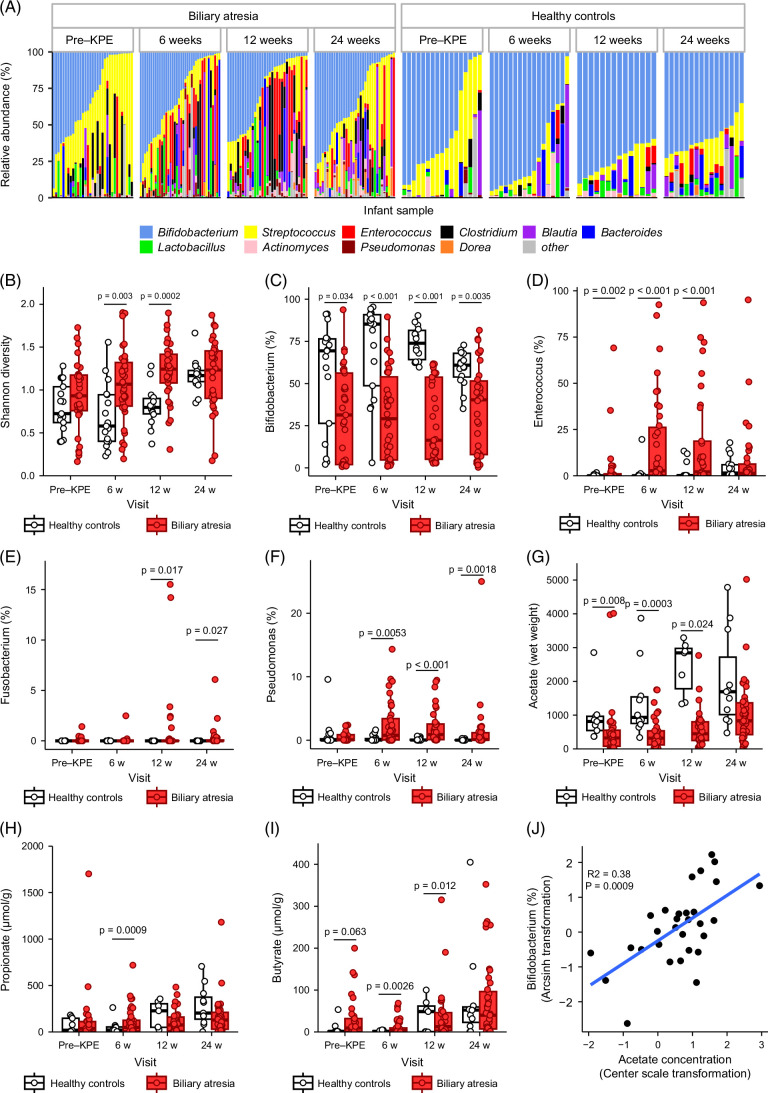
Longitudinal fecal microbiota and SCFA composition comparison between BA and healthy infants. Stacked bar plots (A) showing the 10 most abundant genera in individual BA infants and age-matched healthy controls at pre-KPE, 6 weeks, 12 weeks, and 24 weeks after KPE. (B) Longitudinal Inverse Simpson diversity in BA and age-matched HC infants. Relative abundance comparison of (C) *Bifidobacterium*, (D) *Enterococcus*, (E) *Fusobacterium*, and (F) *Pseudomonas* between BA and healthy infants. Fecal (G) acetate, (H) propionate, and (I) butyrate concentrations in BA and age-matched HC infants. Only significant *p* values included in bar chart comparisons. (J) Significant correlation between *Bifidobacterium* abundance and acetate concentration at 24 weeks in BA infants. The blue line on the scatter plot indicates the line of best fit determined by linear regression. Abbreviations: BA, biliary atresia; HC, healthy control; KPE, Kasai portoenterostomy; SCFA, short-chain fatty acids.

Figure 3A illustrates a marked difference in the distribution of the top 10 genera between BA and HC infants at each time point. Clustering samples, using NMDS, highlighted distinct clustering between BA and healthy infants at all time points (PERMANOVA; 6w-post-KPE, *R* ^2^ = 0.11, *p* < 0.001; 12w-post-KPE, *R* ^2^ = 0.17, *p* < 0.001; 24w-post-KPE, *R* ^2^ = 0.08, *p* = 0.002; Supplemental Figures S2A–C, http://links.lww.com/HC9/B57). Microbiota diversity measured using Shannon and Inverse Simpson diversity was higher at 6w- and 12w-post-KPE in BA compared to healthy infants but similar between groups at 24w-post-KPE (Figure 3B, Supplemental Figure S2D, http://links.lww.com/HC9/B57). A lower abundance of *Bifidobacterium* in BA versus HC infants was demonstrated at all time points (Figure 3C), accompanied by a consistently higher relative abundance of *Enterococcus*, *Pseudomonas*, and *Clostridium* at 6w- and 12w-post-KPE, *Fusobacterium* at 12w-post-KPE (Figures 3D–F, Supplemental Figure S2E, http://links.lww.com/HC9/B57), and *Haemophilus* and *Actinomyces* (Supplemental Figures S2F, G, http://links.lww.com/HC9/B57) at 6w-post-KPE.

Fecal SCFA profiles differed significantly between BA and HC infants at each time point. Acetate concentration was significantly lower at 6w- (*p* = 0.003) and 12w-post-KPE (*p*=0.024) in BA compared to healthy infants (Figure 3H). The concentrations of propionate and butyrate were strikingly higher in BA infants at 6w-post-KPE (Figures 3H, I). The Pearson correlation analysis was used to identify associations between individual genus abundance and fecal SCFA concentrations within the BA infant group (Supplemental Figures S3A–D, http://links.lww.com/HC9/B57). Significant microbiota-SCFA correlations were noted at 24w-post-KPE; notably, *Bifidobacterium* revealed a strong positive correlation with acetate (*r* = 0.56, *p* = 0.017) (Figure 3J).

### Association between fecal microbiota and SCFA profiles and primary clinical outcome measures

To explore the clinical relevance of fecal microbiota in BA infants, fecal microbiota composition was compared between favorable and nonfavorable clinical outcome measures. Baseline characteristics (demographics, diet, and pharmacotherapy) were similar between outcome groups (Supplemental Tables S4 and S6, http://links.lww.com/HC9/B57). Dietary and pharmacotherapy characteristics for longitudinal time points are represented in Supplemental Tables S5 and S7, http://links.lww.com/HC9/B57.

### Jaundice clearance

**FIGURE 4:**
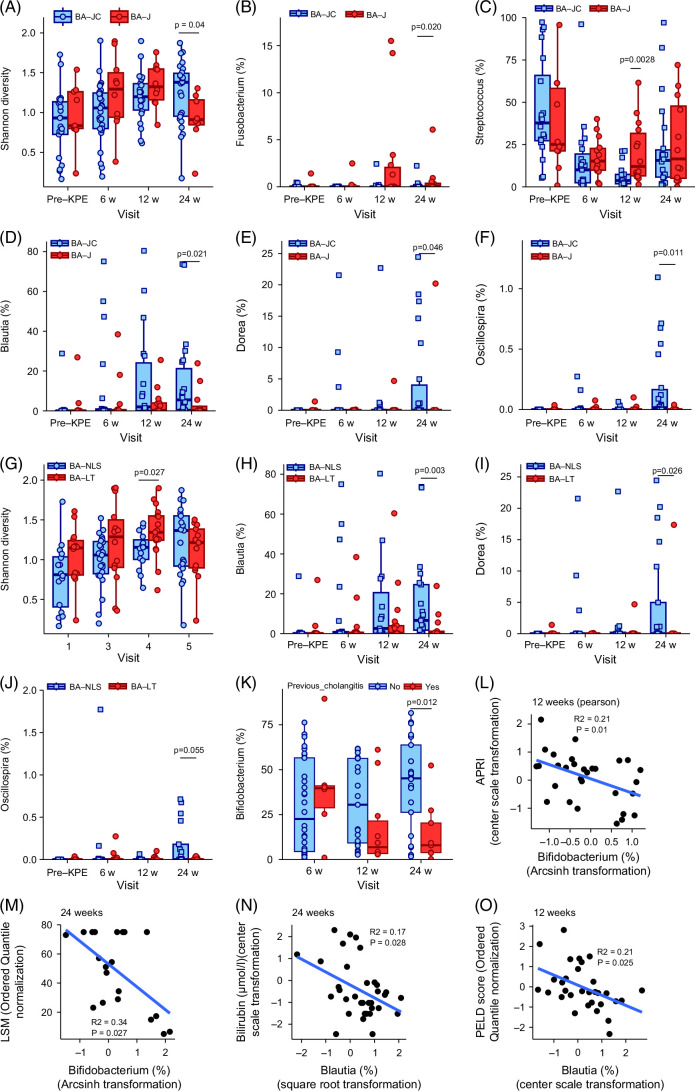
Fecal microbiota and SCFA associations with clinical outcomes. (A) Shannon diversity of fecal microbiota between BA-J and BA-JC groups. Relative abundance comparison of (B) *Fusobacterium*, (C) *Streptococcus*, (D) *Blautia*, (E) *Dorea*, and (F) *Oscillospira* between BA-J and BA-JC groups. (F) Shannon diversity of fecal microbiota between BA-NLS and BA-LT groups. Relative abundance comparison of (G) *Blautia*, (H) *Dorea*, (I) *Oscillospira* between BA-NLS and BA-LT groups. Correlation between (J) APRI versus *Bifidobacterium* at 12 weeks. (K) Correlation between LSM versus *Bifidobacterium* at 24 weeks. Only significant *p* values included in bar chart comparisons. (L) Correlation between Bilirubin versus *Blautia* at 12 weeks. (M) Correlation between PELD versus *Blautia* at 12 weeks. The blue line on scatter plots (I–M) indicates the line of best fit determined by linear regression. (N) *Bifidobacterium* relative abundance at 6, 12, and 24 weeks in BA infants, based on previous cholangitis. Abbreviations: BA, biliary atresia; HC, healthy control; JC, jaundice clearance; KPE, Kasai portoenterostomy; LSM, liver stiffness measurement; LT, liver transplantation; NLS, native liver survival; PELD, Pediatric End-Stage Liver Disease; SCFA, short-chain fatty acids.

Shannon diversity and Inverse Simpson diversity were higher at 12w-post-KPE in BA-J versus BA-JC groups (Figure 4A, Supplemental Figure S4A, http://links.lww.com/HC9/B57). *Fusobacterium* abundance (*p* = 0.009) was higher at 24w-post-KPE and *Streptococcus* higher (*p* = 0.003) at 12w-post-KPE in BA-J compared to BA-JC groups (Figures 4B, C). Relative abundance of *Blautia* (*p* = 0.021), *Dorea* (*p* = 0.046), and *Oscillospira* (*p* = 0.011) was lower at 24w-post-KPE in BA-J compared to BA-JC groups (Figures 4D–F).

### NLS

Shannon diversity and Inverse Simpson diversity were higher at 12w-post-KPE in BA-LT compared to BA-NLS groups (Figure 4G, Supplemental Figure S4B, http://links.lww.com/HC9/B57). *Blautia* (*p* = 0.003) and *Dorea* (*p* = 0.026) abundance was lower (Figures 4H, I), and *Oscillospira* tended to be lower (*p* = 0.055) (Figure 4J) at 24w-post-KPE in BA-LT compared to BA-NLS groups. No differences between SCFA abundance and primary clinical outcome measures were revealed.

### Association between fecal microbiota and cholangitis

Fecal microbiota was compared between BA infants who had experienced “one or more episode(s) of cholangitis” versus “no cholangitis” between each time point. Sixteen percent (n = 6), 30% (n = 10), and 27% (n = 9) developed an episode(s) of cholangitis between “KPE to 6w-post-KPE,” “6w- to 12w-post-KPE,” “12w- to 24w-post-KPE,” respectively. Differential abundance testing identified lower *Bifidobacterium* (*p* = 0.012) abundance by 24w-post-KPE in infants experiencing cholangitis in the preceding time period (Figure 4K). No difference in SCFA profile was revealed between cholangitis and no cholangitis groups. No difference in fecal microbiota/SCFA profiles in infants who subsequently developed an episode(s) of cholangitis was observed (data not shown).

### Correlation between fecal microbiota and SCFA profiles and liver disease severity and biomarkers of liver fibrosis

Pearson correlation analysis was used to determine associations between the top 10 abundant genera and parameters of liver disease severity (PELD and TB) and liver fibrosis (AST-to-platelet ratio index and liver stiffness measurement) (Supplemental Figures S5A–D, http://links.lww.com/HC9/B57). Notably, *Bifidobacterium* inversely correlated with fibrosis markers (AST-to-platelet ratio index, *r* ^2^ = 0.21, *p* = 0.01; liver stiffness measurement, *r* ^2^ = 0.34, *p* = 0.027) (Figure 4LM) and *Blautia* inversely correlated with parameters of liver disease severity (TB, *r* ^2^ = 0.17, *p* = 0.028; PELD, *r* ^2^ = 0.21, *p* = 0.025) (Figures 4N, O; Supplemental Figure S5E, http://links.lww.com/HC9/B57). No correlation between SCFAs and disease severity or fibrosis was revealed.

## DISCUSSION

Exploring the role of the gut microbiota and its potential downstream effects in early BA could uncover a crucial modifiable pathway for early BA therapeutics. In this novel longitudinal study, pre- and post-KPE dysbiosis (increased diversity, expansion of pathobionts, and depletion of beneficial microbiota) and SCFA disturbance in BA infants were described. Importantly, dysbiosis was associated with relevant BA clinical outcomes.

A notable divergence in beta diversity, indicating the composition of the gut microbiota, was revealed when comparing the BA microbiota against the microbiota of healthy infants.^18^ BA microbiota was characterized by the elevation of potentially harmful bacteria (referred to as “pathobionts”; *Enterococcus*, *Clostridium*, *Fusobacterium*, and *Pseudomonas*) and a reduction in beneficial bacteria (*Bifidobacterium*). The expansion of pathobionts has been reported in studies of adult cholestasis and cirrhosis, as well as recent BA studies, with *Bifidobacterium* depletion being increasingly recognized in BA.^8^,^9^,^10^,^11^,^12^,^13^,^14^,^15^,^16^

The similarity between gut microbiota profiles between pre-KPE BA and non-BA cholestatic infants suggests cholestasis as a microbiota-shaping factor. Germ-free mouse models for biliary disease have highlighted an association between the gut microbiota and cholestasis.^19^,^20^ Gut microbiota interact with bile acids through exerting bile salt hydrolase activity, which deconjugates primary bile acids and, through 7-alpha-hydroxylation, converts primary to secondary bile acids. In turn, bile acids influence microbial composition through mechanisms such as microbial gene regulation, immune modulation, and regulation of gut barrier integrity.^5^ This intricate microbiota-bile acid interplay was illustrated in an adult cirrhosis cohort, where a positive correlation between *Ruminococcaceae* (7-alpha-hydroxylating bacteria) and deoxycholic acid (secondary bile acid) was demonstrated. In BA, serum bile acid profiles have been explored as a discriminatory tool between BA and non-BA infantile cholestasis, but increasingly, microbiota studies are incorporating fecal bile acid analysis as a functional pathway.^9^,^10^ One BA study demonstrated a positive correlation between *Enterococcus* and secondary bile acid derivatives,^10^ potentially posing a functional pathway for this pathobiont in BA.

A striking feature of this study was the markedly increased alpha diversity in the gut microbiota in the early post-KPE period and its association with poor jaundice clearance and LT. Beta diversity, incorporating pathobiont expansion (*Fusobacterium* and *Streptococcus*) and depletion of beneficial microbiota “probiotics” (*Blautia*, *Dorea*, and *Oscillospira*) were also linked to these poorer outcomes. *Streptococcaceae* and *Fusobacteriaceae* have been similarly associated with cholestatic disease and disease severity in cirrhosis in adult and pediatric cohorts^7^,^9^; however, unraveling the extent to which liver disease is propelling dysbiosis or vice versa is challenging in the clinical setting. Human isolates of *Fusobacterium varium* ^21^ have been shown to induce colonic mucosal erosion in mice by rectal enema, postulated as a mechanistic link in inflammatory bowel disease. Surprisingly, *Enterococcus* expansion was not associated with clinical outcomes, potentially highlighting more complex downstream interactions rather than linear genus-outcome correlation, requiring further interrogation.

The link between “probiotic” depletion and clinical outcomes is intriguing and could play a crucial role in the development of future BA therapeutics. *Blautia* inversely correlated with liver disease severity parameters. *Blautia* depletion has been described in adult cirrhosis cohorts, with its beneficial interaction with bile acid metabolism, SCFA production, and immunomodulatory potential^22^ influencing host interactions. *Bifidobacterium,* a Gram-positive anerobic genus, which is dramatically depleted after KPE, although not directly associated with clinical outcomes, is inversely correlated with fibrosis markers, potentially highlighting an immune role for this genus in BA. *Bifidobacterium* is well known to confer positive health benefits to the human host, namely through its antibacterial activity,^23^ immunomodulatory potential, stabilization of the intestinal barrier integrity,^24^ and bile acid metabolism through its ability to secrete bile salt hydrolase.^25^ Depletion of *Bifidobacterium* in infancy has been linked to numerous conditions, including necrotizing enterocolitis^26^ and poorer neurodevelopmental outcomes.^27^ *Bifidobacterium* play a critical role in fermenting human milk oligosaccharides (HMOs), nondigestible carbohydrates found in breastmilk; hence, a symbiotic relationship between breastmilk-*Bifidobacterium* exists.^28^ Although higher microbiota diversity is often linked with a “healthy state,” in early infancy, a low diverse, *Bifidobacterium*-dominant signature^28^ is associated with health. Therefore, the substantial decline in breastfeeding rates following KPE likely contributes to *Bifidobacterium* depletion, enrichment of pathobionts, and higher diversity observed in BA infants.

While interactions between dietary factors, cholestasis, and the microbiota are likely to play pivotal roles in BA dysbiosis, the significant changes observed in gut microbiota diversity and composition during the early postsurgical period prompt inquiries into the potential involvement of standard postoperative pharmacotherapy. Prophylactic antibiotics are part of post-KPE protocols worldwide, although their impact on BA clinical outcomes remains uncertain. Antibiotic-associated dysbiosis has been revealed in several infant studies.^29^ In our study, *Bifidobacterium* abundance was significantly reduced following cholangitis infection, raising the possibility of antibiotic-associated *Bifidobacterium* depletion.^29^ Corticosteroids and choleretic administration after KPE pose uncertain clinical benefits^30^ and have also been shown to influence microbial patterns.^31^,^32^ Therefore, gaining a deeper comprehension of the interplay between BA therapies and microbiota patterns can facilitate the development of more precise mechanistic pathways and refine clinical practice.

The production of SCFAs has surfaced as a pivotal microbiota-downstream pathway. This study, to our knowledge, is the first to examine SCFAs alongside BA gut microbiota. SCFAs, including acetate, propionate, and butyrate, represent the primary end-products of the fermentation of nondigestible carbohydrates exclusively by the gut microbiota. SCFAs influence essential host interactions, such as immune regulation, gut barrier integrity maintenance, and antimicrobial properties (including lowering pH to inhibit pathogens).^33^ In this study, fecal acetate concentration was significantly reduced in pre-KPE BA infants and further exacerbated after KPE. Acetate is the most abundant SCFA in healthy infants, suggesting an evolutionary contribution to infant health. *Bifidobacterium* is a dominant acetate-producing bacterium in infancy; hence, its depletion in BA potentially influences acetate depletion. Microbiota-SCFA correlation analysis did reveal a positive correlation between *Bifidobacterium* and acetate by 24w-post-KPE. The absence of a *Bifidobacterium*-acetate association at earlier time points may highlight insufficient substrate (eg, breastmilk) or overall *Bifidobacterium* abundance to exert its functional capacity.

Animal models have highlighted the immunomodulatory and antimicrobial properties of acetate, including inhibition of lipopolysaccharide-induced TNFa secretion.^34^ An association between reduced stool acetate at 3 months of age^35^ and subsequent allergic disease suggests a role for early acetate-mediated developmental immune priming. Hence, downstream implications of acetate depletion in BA fibro-inflammatory pathways require further interrogation. In contrast to acetate reduction, butyrate abundance was higher in BA infants at early post-KPE time points. Butyrate is the preferred fuel used for colonocyte proliferation and regulates gut epithelial barrier integrity through coordinated regulation of the tight junction proteins.^36^ Butyrate-producing microbiota (eg, *Ruminoccocus* and *Clostridales*) are reduced in adult cirrhosis cohorts, and butyrate has been inversely correlated with portal hypertension and inflammatory mediators^37^; however, the role of butyrate in infancy has not been well-defined.

Microbiota-modulatory clinical trials are showing promise in adult liver disease. Several randomized, placebo-controlled clinical trials have revealed probiotic efficacy in various liver diseases, and clinical trials on fecal microbiota transplantation (FMT) have demonstrated safety and favorable changes in gut microbiota composition and function in alcohol-associated hepatitis and HE, with preclinical and pilot data suggesting a role for FMT in nonalcoholic liver disease and primary sclerosing cholangitis.^38^ Few studies have explored the role of probiotics in BA, and only culture-dependent techniques have been used. One study demonstrated similar efficacy in cholangitis prevention between prophylactic *Lactobacillus* casei rhamnosus (probiotic) versus neomycin^39^ in jaundice-free BA patients (0–3 y-old) and beneficial microbiota shifts in BA infants after LT have been demonstrated.^40^ A recent position paper on the use of probiotics in preterm infants to prevent necrotizing enterocolitis supports *Bifidobacterium* prophylaxis.^41^ The findings of this latest study suggest the need for additional exploration into the potential benefits of pre- and probiotic prophylactic supplementation during the early post-KPE period and in cholangitis management.

This study’s strength lies in its comprehensive characterization of the fecal microbiota, coupled with paired fecal SCFA analysis in BA, using both cholestatic patients and HCs. Nonetheless, certain limitations should be acknowledged. First, while the rationale behind selecting exclusively breastfed, pharmacotherapy-naïve infants as HCs has been discussed, the divergence of dietary and pharmacotherapy characteristics between cohorts are significant confounders. Future studies could consider incorporating formula-fed, antibiotic-exposed infants alongside the “gold-standard” control arm to mitigate these confounders. Second, a non-BA CC cohort was only available at the first time point due to the challenges of obtaining CCs matched for microbiota-shaping factors at the longitudinal time points. Third, although 16S rRNA gene amplicon sequencing data provide valuable genus-level profiling in a relatively new disease area, combining it with shotgun metagenomics could offer species/strain data and potential functional signatures for future studies. Lastly, dietary data collection was limited to 3-day milk diaries, which may introduce recall bias, and extensive pharmacotherapy administration (eg, antireflux medications) was not examined due to small subcohorts.

## CONCLUSIONS

The microbiota and SCFA profiles in BA represent a notable departure from healthy microbiota patterns, especially following Kasai surgery. Microbial disturbance after Kasai has been linked to clinical outcomes, highlighting the importance of scrutinizing post-Kasai protocols in mitigating their potential contributions and exploring strategies to refine these protocols, such as the incorporation of prebiotics or probiotics.

## Supplementary Material

## Data Availability

Data, analytical methods, and study materials are available to other researchers under specific requests. The data collected for the study, including anonymous individual participant data and the data dictionary defining each field in the set, will be made available to others for scientific purposes. The data and related documents, including analytical methods and study materials, will be available after the publication date on specific request to vjain@nhs.net, with a signed data access agreement and restriction of publication without the authors’ consent. Vandana Jain and Anil Dhawan conceptualized the study. Anil Dhawan supervised and mentored Vandana Jain. Vandana Jain oversaw the investigation. Vandana Jain was responsible for acquiring the resources. Vandana Jain was responsible for sample collection. Emma C. Alexander and Charlotte Burford contributed to sample collection and processing. Vandana Jain, Holly Acford-Palmer, Iliana R. Serghiou, Nancy M.Y. Teng, Konstantinos Gerasimidis, Michael Logan, and Konstantina Zafeiropoulou were involved in sample processing and analysis. Matthew J. Dalby and Raymond Kiu carried out a bioinformatic analysis. Mark Davenport contributed to acquiring resources and data interpretation. Anita Verma and Konstantinos Gerasimidis contributed to data interpretation. Matthew J. Dalby, Vandana Jain, and Charlotte Burford participated in the statistical analysis. Matthew J. Dalby was responsible for the visualizations of data. Vandana Jain and Matthew J. Dalby wrote the initial draft. Vandana Jain, Anil Dhawan, Matthew J. Dalby, and Lindsay J. Hall edited and reviewed the subsequent drafts. All authors reviewed and commented on subsequent drafts. Vandana Jain and Matthew J. Dalby accessed and verified the data. Joint BSPGHAN/CLDF charity fund. Konstantinos Gerasimidis is on the speakers’ bureau for Nutricia Danone and Nestle. Anil Dhawan consults and owns stock in BitBio and Aspect Bio. He consults for Astellas. The remaining authors have no conflicts to report.
