# Supplementary material for: Association of gut microbiota and gut metabolites and adverse outcomes in biliary atresia: A longitudinal prospective study
Source: Hepatol Commun. 2024 Oct 17;8(11):e0550. doi: 10.1097/HC9.0000000000000550 (PMC11495700; doi:10.1097/HC9.0000000000000550)
Supplement: Supplementary file 1 [file hc9-8-e0550-s001.docx]

Supplementary Material: Contents

[**Supplementary Methods** 3](#_Toc170338104)

[Clinical BA Protocol 3](#_Toc170338105)

[Transient Elastography (TE) 3](#_Toc170338106)

[Scoring systems 4](#_Toc170338107)

[Cholangitis definitions 4](#_Toc170338108)

[16S rRNA gene amplicon sequencing of faecal samples and sequence analysis 5](#_Toc170338109)

[**Supplementary Results** 7](#_Toc170338110)

[Characteristics of pre-KPE BA, Healthy control and Cholestatic Control cohorts 7](#_Toc170338111)

[Supplementary Table 1 (S1). Comparison of pre-KPE baseline characteristics between (a) BA-JC vs BA-J groups and (b) BA-NLS vs BA-LT groups, for faecal microbiota analysis. 8](#_Toc170338112)

[Supplementary Table 2 (S2). Comparison of dietary and pharmacotherapy characteristics between BA-NLS vs BA-LT groups at (i) 6w- (ii) 12w- (iii) 24w-post-KPE, for faecal microbiota analysis. 9](#_Toc170338113)

[Supplementary Table 3 (S3). Comparison of pre-KPE baseline characteristics between (a) BA-JC vs BA-J groups and (b) BA-NLS vs BA-LT groups, for faecal SCFA analysis. 10](#_Toc170338114)

[Supplementary Table 4 (S4). Comparison of dietary and pharmacotherapy characteristics between BA-NLS vs BA-LT groups at (i) 6w- (ii) 12w- (iii) 24w-post-KPE, for faecal SCFA analysis. 11](#_Toc170338115)

[Supplementary Figure 1 (SF1). Faecal microbiota and SCFA comparison between pre-KPE BA, HC and CC cohorts 12](#_Toc170338116)

[13](#_Toc170338117)

[13](#_Toc170338118)

[Supplementary figure 2 (SF2). Longitudinal microbiota composition comparison between BA and healthy infants. 13](#_Toc170338119)

[Supplementary Figure 3 (SF3). Correlation analysis between faecal microbiota and SCFAs 14](#_Toc170338120)

[Supplementary Figure 4 (SF4). Faecal microbiota associations with clinical outcomes 15](#_Toc170338121)

[Inverse Simpson Diversity comparison between (A) BA-J vs BA-JC groups and (B) BA-NLS and BA-LT groups. 15](#_Toc170338122)

[Supplementary Figure 5 (SF5). Correlation analysis between faecal microbiota and liver disease severity and fibrosis parameters 17](#_Toc170338123)

# **Supplementary Methods**

## Clinical BA Protocol

All BA infants underwent pre-operative bowel decontamination, consisting of metronidazole, lactulose and gentamicin, 48 hours prior to KPE procedure. Type of anatomic BA (Isolated Type III, syndromic, cystic) was characterised macroscopically. Standard post-operative antibiotics included intravenous (IV) piperacillin/tazobactam and gentamicin, for minimum of 5 and 2 days, respectively. Oral cephalexin prophylaxis, was commenced after completion of IV antibiotics, for one month. As per departmental protocol, prednisolone was administered on discharge from hospital, if infants fulfilled the following criteria; >37 weeks gestation, cytomegalovirus (CMV) IgM negative, infection-free, less than 70 days of age at KPE. Prednisolone was commenced at 5mg/kg/day, and tapered over a 6-7 week period, and then stopped. Anti-reflux medication, either ranitidine or omeprazole, was administered as gastro-protective agents, during the steroid course. CMV IgM positive infants, were commenced on antiviral treatment (either IV ganciclovir or oral valganciclovir). Other standard medications included vitamins (multivitamin, E, K) and choleretics (phenobarbitone, ursodeoxycholic acid).

## Transient Elastography (TE)

TE, using the Fibroscanner 502 (Echosens, Paris, France), was performed on BA infants at all time-points. As per the manufacturers recommendation ^1^, the 5mm child “S1” probe was placed in the 10th intercostal space in the right mid axillary line for the liver. A valid examination was defined as 10 measurements with a total success rate ≥ 90% and the ratio of interquartile range to median value was <30%. The median value of the 10 measurements, was reported as the liver stiffness measurement (LSM; kPa). TE measurements were not possible in infants with gross ascites.

## Scoring systems

The paediatric liver disease severity scoring system, Paediatric End-Stage Liver Disease (PELD), was calculated at each time-point for all BA infants ^2^.

PELD Score = 10 x (0.480 x ln(bilirubin) + 1.857 x ln(INR) - 0.687 x ln(albumin) + 0.436 (if patient under 12 months) + 0.667 (if weight or height <-2 SD).

The biomarker for fibrosis, Aspartate Aminotransferase-to-Platelet Ratio index (APRi), was calculated at each time-point ^3^ for all BA infants.


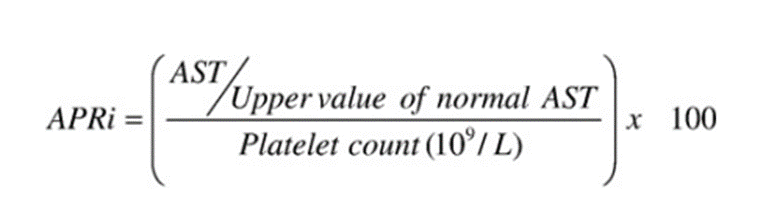


## Cholangitis definitions

Two cholangitis definitions, were used in this study, adapted from the Tokyo 2018 guidelines ^4,5^, developed for adults.

- *Likely cholangitis,* defined as fever (>38C) for 24 hours with or without positive blood culture AND no other source of infection detected AND abnormal inflammatory markers (either CRP>5 mg/L or WCC < 4 x10^9^ or > 10 x10^9^) AND an increase in TB levels.
- *Suspected cholangitis,* defined as (i) sharp increase in TB levels with or without fever AND/OR abnormal inflammation markers and NO other evidence of extrahepatic infection OR (ii) fever and raised inflammatory markers with no increase in TB levels and NO other evidence of extrahepatic infection.

## 16S rRNA gene amplicon sequencing of faecal samples and sequence analysis

16S rRNA gene amplicon sequencing of faecal samples 16S rRNA region (V1-V2) primers were used for library construction. This set of primers allowed the amplification of one 16S rRNA gene sequence ng library containing 96 different samples. PCR conditions used were: cycle of 94°C 3 min and 25 cycles of 94°C for 45 s, 55°C for 15 s and 72°C for 30 s. Sequencing of the 16S rRNA gene libraries was performed using Illumina MiSeq platform with 300 bp paired end reads. Sequencing raw reads (FASTQ) were firstly merged using PEAR v0.9.6^6^, followed by quality filtering via scripts within QIIME v1.9.1^7^ split_libraries_fastq.py, chimera identification and removal via identify_chimeric_seqs.py and filter_fasta.py respectively. Operation taxonomic unit (OTU) clustering method was used to assign taxonomy to sequence reads based on SILVA_132 reference database^8^. This OTU picking step was run using QIIME open reference script pick_open_reference_otus.py. The BIOM output files were visualised on MEGAN6^9^.

The number of reads in sequenced samples ranged from 1,888 to 249,736 with a median value of 33,813. The median number of genera detected across all samples was 13. Visualization of genera number by sampling depth using rarefaction curves generated in the vegan package in R indicated that the sequencing depth was adequate in all samples due to the small number of genera present. To account of varying sequencing depth between samples the data was normalised using Variance Stabilizing Transformation in the DESeq2 package in R^10^.

# **Supplementary Results**

## Characteristics of pre-KPE BA, Healthy control and Cholestatic Control cohorts

Non-BA cholestatic cohort

The non-BA cholestatic cohort comprised the following; prematurity/parenteral nutrition (5) alpha-1-antitrypsin deficiency (4), Alagille syndrome (2), inspissated bile syndrome (2), ischemic injury (1), idiopathic neonatal hepatitis (1), progressive familial intrahepatic cholestasis Type II (1), neonatal sclerosing cholangitis (1), hyperinsulinism (1), Niemann Pick Type C (1), ABO incompatibility (1).

| **Baseline characteristics** | **Sub-Cohort for faecal microbiota  analysis** | | | **BA vs HC p** | **BA vs CC p** | **CC vs HC p** |
| --- | --- | --- | --- | --- | --- | --- |
|  | **BA**  **n=33** | **HC**  **n=17** | **CC**  **n=19** |  |  |  |
| **Demographics** |  | | | | | |
| Age at time-point (days),  median (IQR) | 49  (34, 56) | 38  (33, 50) | 49  (30, 76) | 0.1 | 0.5 | 0.3 |
| Mode of birth delivery (vaginal) | 19 (58) | 11 (65) | 11 (58) | 0.6 | 0.9 | 0.7 |
| Gender (M) | 22 (67) | 8 (47) | 8 (42) | 0.9 | 0.2 | 0.3 |
| Prematurity (< 37 weeks) | 2 (6) | 0 (0) | 5 (26) | 0.3 | ***0.04*** | ***0.02*** |
| Birth Weight (Kg)  median (IQR) | 3.09  (2.8, 3.4) | 3.3  (3, 3.6) | 2.6  (1.4, 3.1) | 0.06 | ***0.003*** | ***<0.001*** |
| **Laboratory parameters** |  | | | | | |
| TB (µmol/l)  Median (IQR) | 129  (100, 147) | na | 95  (58, 136) | - | 0.03 | - |
| GGT (IU/L)  Median (IQR) | 359  (242, 697) | na | 270  (88, 454) | - | 0.05 | - |
| **Pharmacotherapy** |  | | | | | |
| UDCA | 18 (55) | 0 (0) | 17 (90) | ***-*** | **0.01** | ***-*** |
| Postnatal antibiotics | 11 (33) | 0 (0) | 15 (79) | ***-*** | **0.002** | ***-*** |
| **Predominant diet from birth** |  | | | | | |
| Exclusively breast-fed | 10 (30) | 11 (100) | 3 (16) | ***<0.001*** | 0.2 | ***<0.001*** |
| Mixed breast/formula-fed | 13 (39) | 0 (0) | 9 (47) | ***-*** | 0.5 | ***-*** |
| Formula-fed | 9 (27) | 0 (0) | 7 (36) | ***-*** | 0.4 | ***-*** |
| **MCT administration** |  | | | | | |
| MCT-fed | 24 (73) | 0 (0) | 13 (68) | ***-*** | 0.7 | ***-*** |

Supplementary Table 1 (S1). Baseline characteristic comparison between pre-KPE BA and age-matched HC and CC cohorts for faecal microbiota sub-cohort.

| **Baseline characteristics** | **Sub-Cohort for faecal SCFA  analysis** | | | **BA vs HC p** | **BA vs CC p** | **CC vs HC p** |
| --- | --- | --- | --- | --- | --- | --- |
|  | **BA**  **(n=34)** | **HC**  **(n=11)** | **CC**  **(n=15)** |  |  |  |
| **Demographics** |  | | | | | |
| Age at time-point (days),  median (IQR) | 47  (32, 57) | 46  (30, 51) | 59  (29, 76) | 1 | 1 | 0.5 |
| Mode of birth delivery (vaginal) | 19 (56) | 6 (55) | 8 (53) | 0.94 | 0.87 | 0.95 |
| Gender (M) | 24 (71) | 8 (73) | 8 (54) | 0.89 | 0.24 | 0.32 |
| Prematurity (< 37 weeks) | 4 (12) | 0 (0) | 4 (27) | 0.23 | 0.23 | 0.11 |
| Birth Weight (Kg)  median (IQR) | 3.08  (2.7, 3.4) | 3.23  (3, 3.7) | 2.8  (1.5, 3.1) | 0.62 | 0.26 | ***0.04*** |
| **Laboratory parameters** |  | | | | | |
| TB (µmol/l)  Median (IQR) | 130  (102, 150) | na | 95  (56, 134) | na | ***0.01*** | na |
| GGT (IU/L)  Median (IQR) | 406  (236, 679) | na | 294  (70, 454) | na | 0.1 | na |
| **Pharmacotherapy** |  | | | | | |
| UDCA | 22 (65) | 0 (0) | 14 (93) | ***<0.05*** | ***0.04*** | ***<0.05*** |
| Postnatal antibiotics | 13 (38) | 0 (0) | 12 (80) | ***0.02*** | ***0.01*** | ***<0.001*** |
| **Predominant diet from birth** |  | | | | | |
| Exclusively breast-fed | 13 (38) | 11(100) | 3 (20) | ***<0.001*** | 0.13 | ***<0.001*** |
| Mixed breast/formula-fed | 11 (32) | 0 (0) | 7 (47) | ***0.04*** | 0.65 | ***0.02*** |
| Formula-fed | 9 (26) | 0 (0) | 6 (40) | ***<0.05*** | 0.5 | ***<0.05*** |
| **MCT administration** |  | | | | | |
| MCT-fed | 26 (76) | 0 (0) | 9 (60) | ***<0.05*** | 0.2 | ***<0.05*** |

Supplementary Table 2 (S2). Baseline characteristic comparison between pre-KPE BA and age-matched HC and CC cohorts for faecal SCFA sub-cohort.

| **Confounder variable** | Faecal SCFA Analysis | | | | | | | | |
| --- | --- | --- | --- | --- | --- | --- | --- | --- | --- |
|  | **6w-post-KPE** | | | **12w-post-KPE** | | | **24w-post-KPE** | | |
|  | **BA  n=43** | **HC  n=12** | **p** | **BA  n=37** | **HC  n=11** | **p** | **BA  n=39** | **HC  n=11** | **p** |
| **Baseline Characteristics** | | | | | | | | | |
| Age at time-point (weeks) | 12.8  (11, 15) | 13.3  (12.9, 13.9) | 0.3 | 19.6  (17.7, 22) | 22.3  (21, 23) | 0.05 | 33  (30, 35) | 35  (32, 36) | 0.1 |
| Mode of birth delivery (vaginal) | 27 (63) | 7 (58) | 0.8 | 21 (57) | 6 (60) | 0.8 | 22 (56) | 7 (64) | 0.6 |
| Gender (M) | 25 (58) | 4 (33) | 0.1 | 21 (57) | 4 (40) | 0.3 | 21 (54) | 5 (46) | 0.6 |
| Prematurity (< 37 weeks) | 5 (12) | 0 (0) | 0.2 | 4 (11) | 0 (0) | 0.3 | 6 (15) | 0 (0) | 0.2 |
| Birth weight (Kg) | 3 (2.7, 3.4) | 3.5 (3.2, 4) | ***0.01*** | 3.1 (2.9, 3.5) | 3.5 (2.9, 3.6) | 0.2 | 3.09 (2.7, 3.5) | 3.3 (2.8, 3.6) | 0.3 |
| **Pharmacotherapy** | | | | | | | | | |
| UDCA | 42 (98) | 0 (0) | ***<0.001*** | 37 (100) | 0 (0) | ***<0.001*** | 38 (100) | 0 (0) | ***<0.001*** |
| Antibiotics between time-points | 43 (100) | 0 (0) | ***<0.001*** | 17 (46) | 0 (0) | ***0.03*** | 20 (54) | 0 (0) | ***0.001*** |
| **Diet** | | | | | | | | | |
| Exclusively breast-fed | 1 (2) | 12 (100) | ***<0.001*** | 2 (5) | 11 (100) | ***<0.001*** | 3 (8) | 11 (100) | ***<0.001*** |
| Mixed breast/  MCT-fed | 8 (19) | 0 (0) | 0.1 | 5 (14) | 0 (0) | 0.2 | 4 (12) | 0 (0) | 0.2 |
| MCT-fed | 42 (98) | 0 (0) | ***<0.001*** | 34 (92) | 0 (0) | ***<0.001*** | 28 (74) | 0 (0) | ***<0.001*** |
| Extensively formula-fed | 0 (0) | 0 (0) | na | 1 (3) | 0 (0) | 0.6 | 8 (24) | 0 (0) | 0.6 |
| Weaned | 0 (0) | 0 (0) | na | 6 (16) | 2 (14) | 0.7 | 36 (95) | 11 (100) | 0.4 |

Supplementary Table 3 (S3). Baseline characteristic comparison between longitudinal BA and age-matched HC and CC for faecal SCFA sub-cohort.

## Supplementary Table 4 (S4). Comparison of pre-KPE baseline characteristics between (a) BA-JC vs BA-J groups and (b) BA-NLS vs BA-LT groups, for faecal microbiota analysis.

Data are n (%) or median (IQR). Statistical significance represented as p<0.05.

| **Baseline characteristics** | **Faecal Microbiota Analysis** | | | | | |
| --- | --- | --- | --- | --- | --- | --- |
|  | **BA-JC n=20** | **BA-J n=10** | **p** | **BA-NLS n=16** | **BA-LT n=17** | **p** |
| **Demographics** |  | | | | | |
| Age at KPE (days) | 53 (43, 61) | 41 (27, 57) | 0.1 | 49 (40, 56) | 49 (31, 58) | 0.5 |
| Mode of birth delivery (vaginal) | 13 (65) | 5 (50) | 0.4 | 11 (69) | 8 (47) | 0.2 |
| Gender (M) | 14 (70) | 5 (50) | 0.2 | 11 (69) | 10 (59) | 0.5 |
| Prematurity (< 37 weeks) | 1 (5) | 1 (10) | 0.6 | 1 (6) | 1 (6) | 0.9 |
| Birth Weight (Kg) | 3.2  (2.8, 3.5) | 3  (2.9, 3.3) | 0.3 | 3.1  (2.7, 3.5) | 3  (2.9, 3.4) | 0.7 |
| **Pharmacotherapy** |  | | | | | |
| UDCA | 11 (55) | 6 (60) | 0.8 | 9 (56) | 9 (53) | 0.8 |
| Postnatal antibiotics | 7 (35) | 3 (30) | 0.8 | 6 (38) | 5 (29) | 0.6 |
| **Baseline Diet** |  | | | | | |
| Exclusively breast-fed | 4 (20) | 4 (40) | 0.2 | 4 (25) | 5 (29) | 0.8 |
| Mixed breast/formula-fed | 10 (50) | 4 (40) | 0.6 | 7 (44) | 8 (47) | 0.8 |
| Formula-fed | 6 (30) | 2 (20) | 0.3 | 15 (31) | 4 (24) | 0.4 |
| MCT-fed | 13 (65) | 9 (90) | 0.1 | 10 (63) | 14 (82) | 0.2 |

## Supplementary Table 5 (S5). Comparison of dietary and pharmacotherapy characteristics between BA-NLS vs BA-LT groups at (i) 6w- (ii) 12w- (iii) 24w-post-KPE, for faecal microbiota analysis.

Data are n (%) or median (IQR). Statistical significance represented as p<0.05. na=no assessment. Comparisons between BA-JC and BA-J groups revealed similar significance values (data not displayed).

| **Baseline Characteristic** | **Faecal Microbiota Analysis** | | | | | | | | |  |
| --- | --- | --- | --- | --- | --- | --- | --- | --- | --- | --- |
|  | **6w-post-KPE** | | | **12w-post-KPE** | | | **24w-post-KPE** | | |  |
|  | **BA-NLS n=23** | **BA-LT n=15** | **p** | **BA-NLS n=15** | **BA-LT n=18** | **p** | **BA-NLS n=21** | **BA-LT n=12** | **p** |  |
|  |  |  |  |  |  |  |  |  |  |  |
| Age (weeks) | 12.4 (10.7, 16) | 13 (10.7, 15) | 1 | 19 (17, 22) | 20 (18, 22) | 0.4 | 32 (30, 35) | 33 (30, 35) | 0.9 |  |
| **Diet** |  | | | | | | | | |  |
| Exclusively breast-fed | 0 (0) | 1 (7) | 0.2 | 0 (0) | 1 (6) | 0.4 | 1 (5) | 1 (8) | 0.7 |  |
| Mixed breast/MCT-fed | 6 (26) | 1 (7) | 0.2 | 5 (33) | 0 (0) | ***0.01*** | 2(10) | 1 (33) | 0.9 |  |
| MCT-fed | 23 (100) | 14 (93) | 0.2 | 13 (87) | 16 (89) | 0.8 | 14 (67) | 10 (83) | 0.3 |  |
| MCT volume |  |  |  |  |  |  |  |  |  |  |
| Exclusively formula-fed | 0 (0) | 0(0) | na | 2 (13) | 1 (6) | 0.4 | 6 (29) | 1 (8) | 0.2 |  |
| Weaned | 0 (0) | 0 (0) | 1 | 1 (7) | 5 (28) | 0.1 | 18 (86) | 11 (92) | 0.6 |  |
| **Pharmacotherapy** |  | | | | | | | | |  |
| Steroid | 20 (87) | 14 (93) | 0.5 | 11 (73) | 17 (94) | 0.09 | 16 (76) | 11 (92) | 0.2 |  |
| Antibiotics | 23 (100) | 15 (100) | 0.4 | 6 (40) | 9 (50) | 0.6 | 9 (43) | 8 (72) | 0.1 |  |
| UDCA | 22 (96) | 15 (100) | 0.4 | 14 (93) | 18 (100) | 0.3 | 20 (95) | 12 (100) | 0.4 |  |

| **Baseline Characteristic** | **Faecal SCFA Analysis** | | | | | |
| --- | --- | --- | --- | --- | --- | --- |
|  | **BA-JC n=20** | **BA-J n=12** | **p** | **BA-NLS n=17** | **BA-LT n=17** | **p** |
| **Demographics** |  | | | | | |
| Age at KPE (days) | 46.5 (33.2, 60.7) | 48 (29, 58) | 0.7 | 46 (33, 54) | 51 (31, 60) | 0.5 |
| Mode of birth delivery (vaginal) | 12 (60) | 7 (58) | 0.9 | 11 (65) | 8 (47) | 0.3 |
| Gender (M) | 15 (75) | 7 (58) | 0.3 | 12 (71) | 12 (71) | 1 |
| Prematurity (< 37 weeks) | 2 (10) | 2 (17) | 0.6 | 2 (12) | 2 (12) | 1 |
| Birth Weight (Kg) | 3.09  (2.7, 3.4) | 3.1  (2.9, 3.5) | 0.4 | 3.1  (2.5, 3.4) | 3.02  (2.9, 3.4) | 0.6 |
| **Pharmacotherapy** |  | | | | | |
| UDCA | 12 (60) | 9(75) | 0.4 | 11 (65) | 11 (65) | 1 |
| Postnatal abx usage | 9 (45) | 3 (25) | 0.3 | 8 (47) | 5 (29) | 0.3 |
| **Diet** |  | | | | | |
| Exclusively breast-fed | 6 (32) | 4 (33) | 0.9 | 6 (38) | 4 (24) | 0.4 |
| Mixed breast/formula-fed | 8 (42) | 5 (42) | 0.9 | 6 (38) | 8 (47) | 0.6 |
| Formula-fed | 13 (68) | 8 (67) | 0.9 | 10 (63) | 13 (76) | 0.4 |
| MCT-fed | 12 (63) | 11 (92) | 0.08 | 10 (62) | 15 (88) | 0.09 |

## Supplementary Table 6 (S6). Comparison of pre-KPE baseline characteristics between (a) BA-JC vs BA-J groups and (b) BA-NLS vs BA-LT groups, for faecal SCFA analysis.

Data are n (%) or median (IQR). Statistical significance represented as p<0.05.

| **Confounder Variable** | **Faecal SCFA Analysis** | | | | | | | | |  |
| --- | --- | --- | --- | --- | --- | --- | --- | --- | --- | --- |
|  | **6w-post-KPE** | | | **12w-post-KPE** | | | **24w-post-KPE** | | |  |
|  | **BA-NLS n=23** | **BA-LT n=20** | **p** | **BA-NLS n=15** | **BA-LT n=22** | **p** | **BA-NLS n=22** | **BA-LT n=17** | **p** |  |
|  |  |  |  |  |  |  |  |  |  |  |
| Age (weeks) | 12.4 (10.7, 15.6) | 13.2 (11, 15) | 0.9 | 19 (17, 22) | 19.5 (18, 22) | 0.7 | 33 (30, 36) | 33 (31, 35) | 0.7 |  |
| **Diet** |  | | | | | | | | |  |
| Exclusively breast-fed | 0 (0) | 1 (5) | 0.3 | 1 (7) | 1 (5) | 0.8 | 2 (9) | 1 (6) | 0.8 |  |
| MCT-fed | 23 (100) | 19 (98) | 0.3 | 14 (93) | 20 (91) | 0.8 | 14 (64) | 14 (82) | 0.1 |  |
| Mixed breast/MCT-fed | 7 (30) | 1 (5) | 0.3 | 5 (33) | 0 (0) | ***0.004*** | 1 (5) | 0 (0) | 0.4 |  |
| Standard Formula-fed | 0 (0) | 0 (0) | na | 0 (0) | 1 (5) | 0.4 | 6 (27) | 1 (6) | 0.1 |  |
| Weaned | 0 (0) | 0 (0) | na | 0 (0) | 6 (27) | ***0.03*** | 21 (96) | 15 (88) | 0.8 |  |
| **Pharmacotherapy** |  | | | | | | | | |  |
| Steroid | 19 (83) | 20 (47) | 0.5 | 11 (73) | 20 (91) | 0.2 | 17 (77) | 15 (88) | 0.4 |  |
| Antibiotic | 23 (100) | 20 (100) | na | 6 (40) | 12 (55) | 0.4 | 10 (46) | 10 (66) | 0.2 |  |
| UDCA | 22 (96) | 20 (100) | 0.3 | 15 (100) | 22 (100) | na | 22 (100) | 22 (100) | na |  |

## Supplementary Table 7 (S7). Comparison of dietary and pharmacotherapy characteristics between BA-NLS vs BA-LT groups at (i) 6w- (ii) 12w- (iii) 24w-post-KPE, for faecal SCFA analysis.

Data are n (%) or median (IQR). Statistical significance represented as p<0.05.. na=no assessment. Comparisons between BA-JC and BA-J groups revealed similar significance values (data not displayed).

**
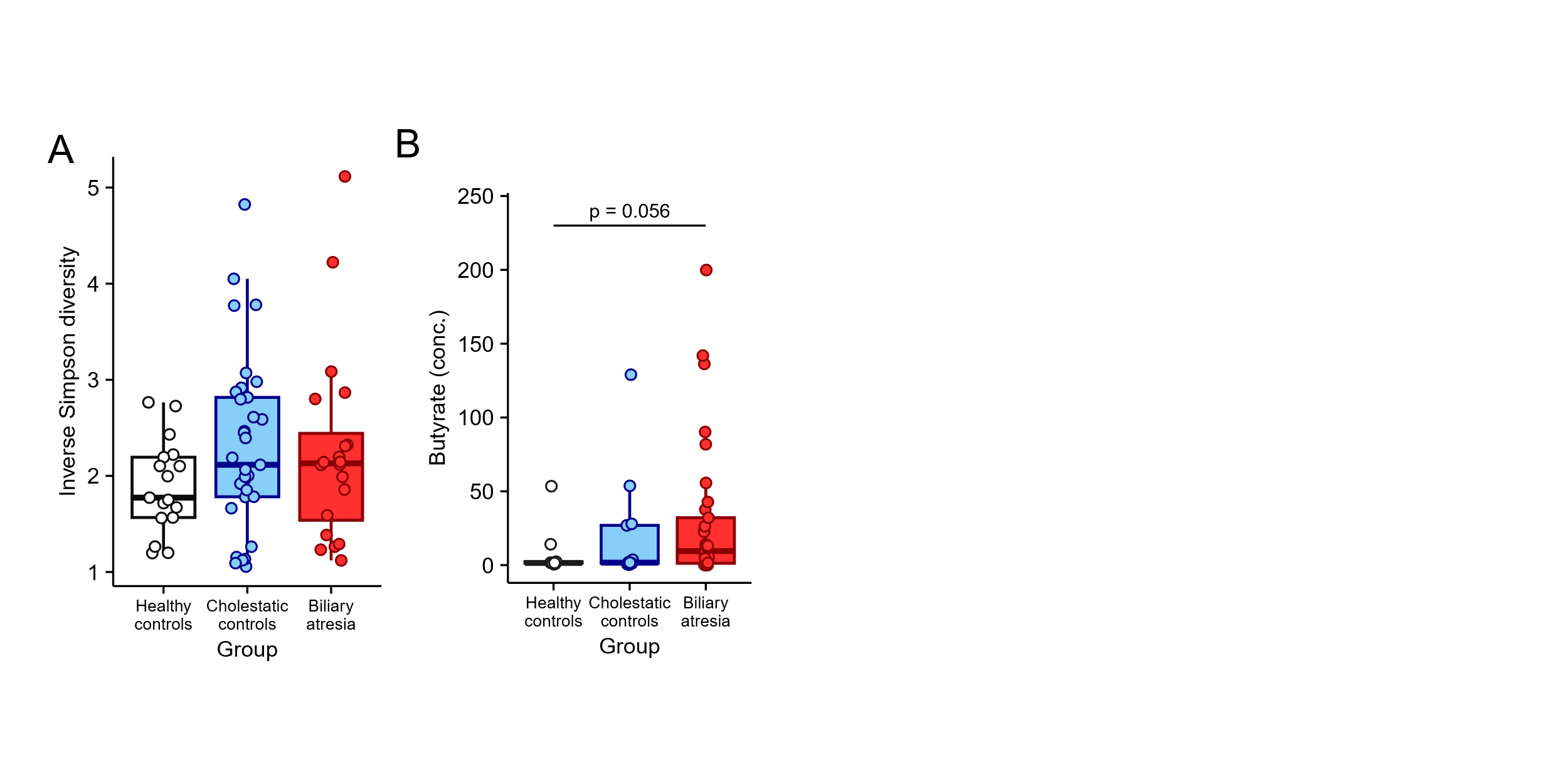
**

## Supplementary Figure 1 (SF1). **Faecal microbiota and SCFA comparison between pre-KPE BA, HC and CC cohorts**

(A) Inverse Simpson diversity in pre-KPE BA, age-matched HC and CC infants. (B) Butyrate concentrations in pre-KPE BA, age-matched HC and CC infants.


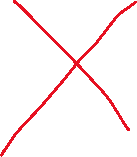


##
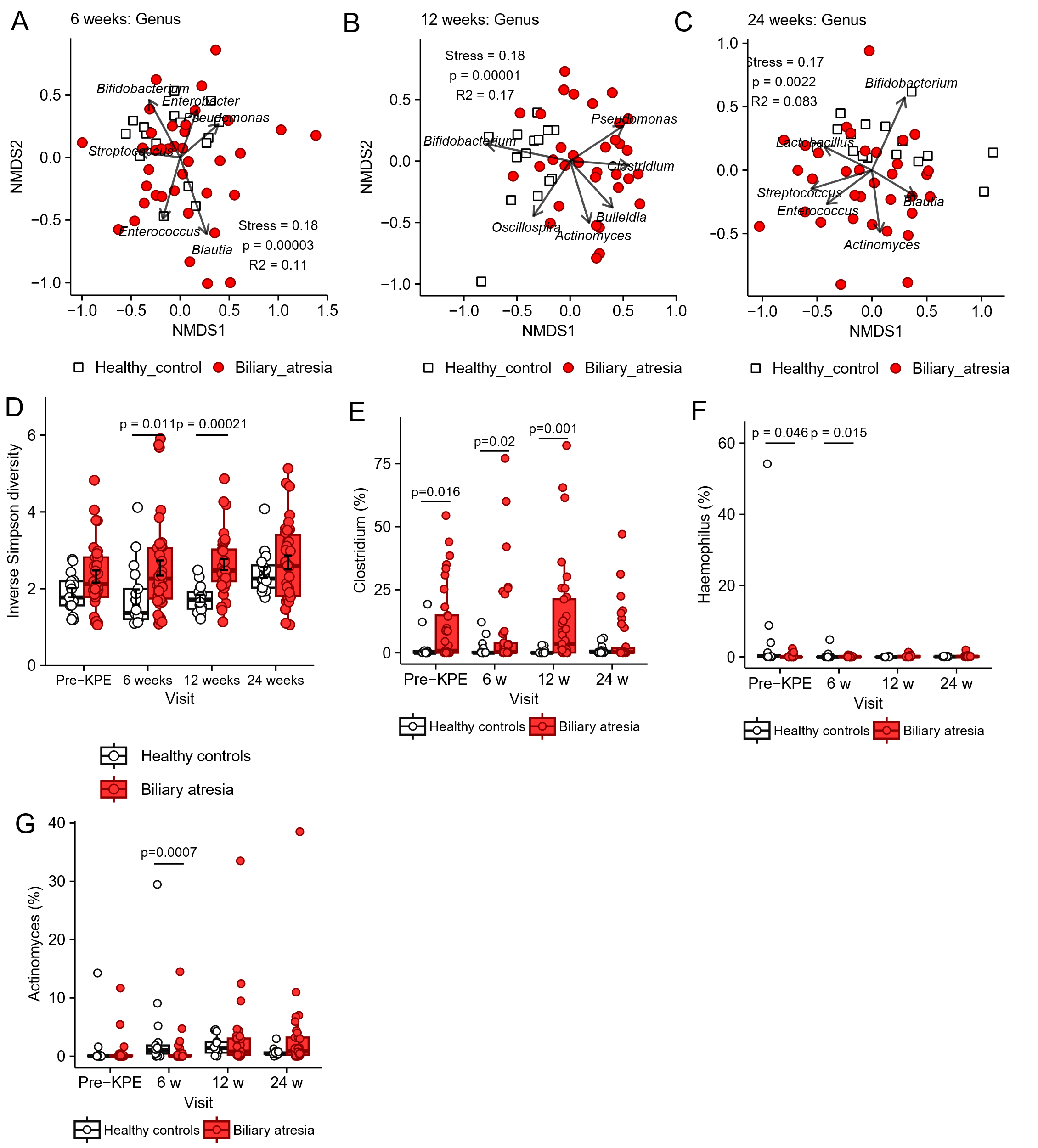


## Supplementary figure 2 (SF2). **Longitudinal microbiota composition comparison between BA and healthy infants.**



NMDS plots showing beta diversity at (A) 6 weeks (B) 12 weeks and (C ) 24 weeks-post KPE. (D) Longitudinal Shannon diversity in BA and age-matched HC infants. Longitudinal relative abundance of (E) *Haemophilus* and (F) *Actinomyces* in BA infants across time-points.

**
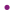
**

**
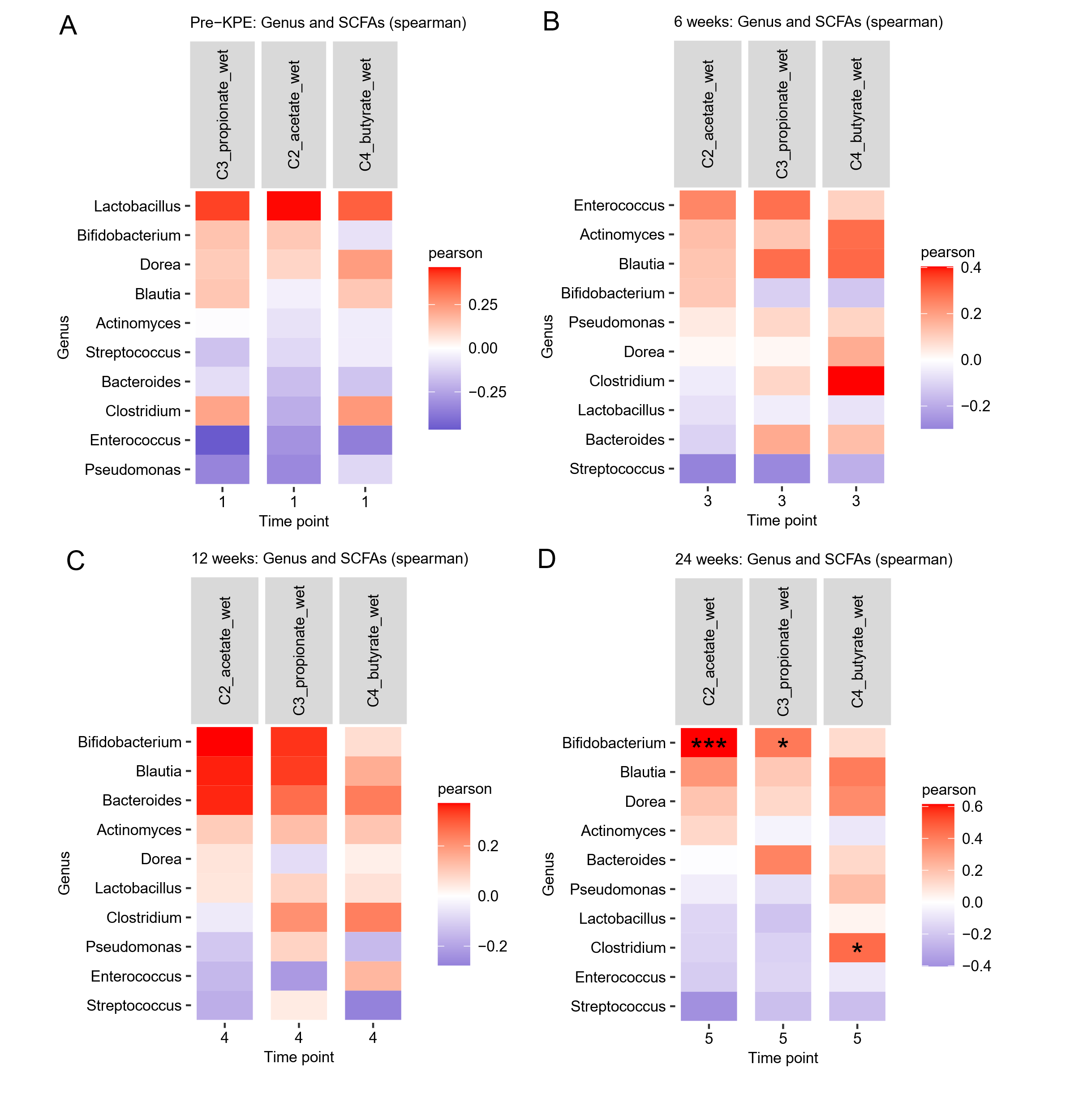
**

## Supplementary Figure 3 (SF3). **Correlation analysis between faecal microbiota and SCFAs**

Pearson correlation between top genera with the top ten most abundant genera and faecal SCFA concentration in (A) pre-KPE and (B) 6 weeks- (C ) 12 weeks- and (D) 24 weeks-post KPE.


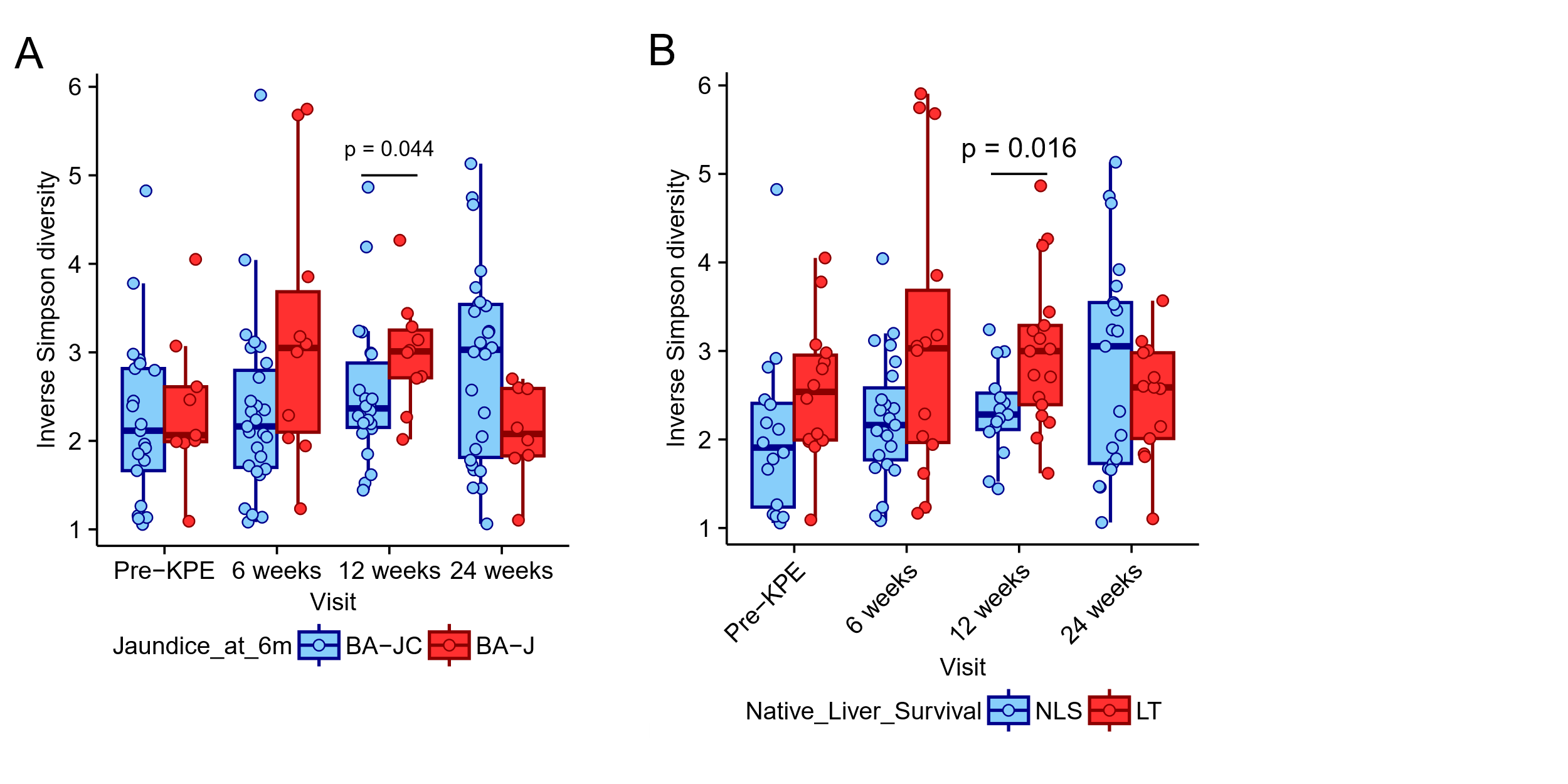


## Supplementary Figure 4 (SF4). **Faecal microbiota associations with clinical outcomes**

## Inverse Simpson Diversity comparison between (A) BA-J vs BA-JC groups and (B) BA-NLS and BA-LT groups.


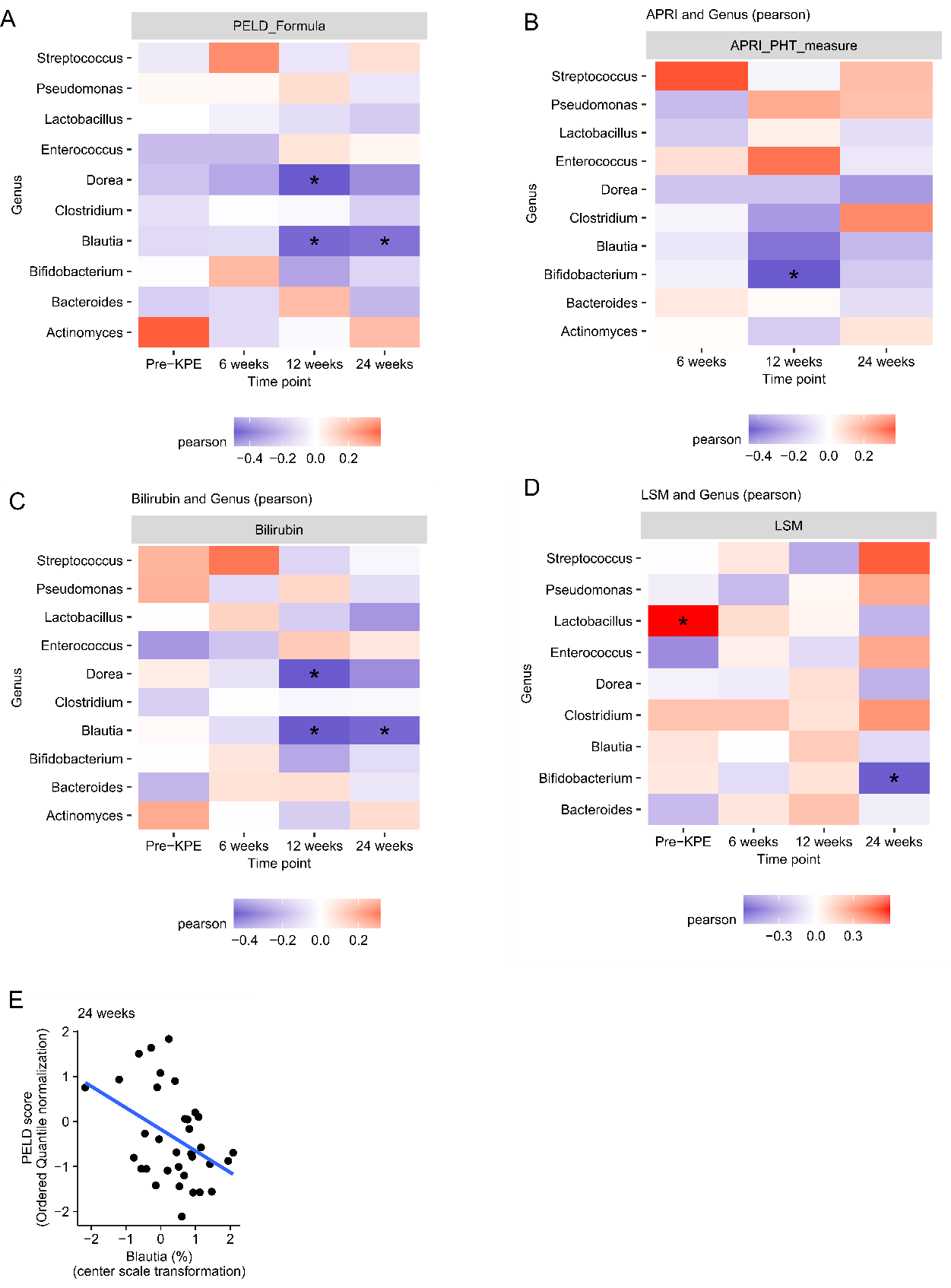


## Supplementary Figure 5 (SF5). **Correlation analysis between faecal microbiota and liver disease severity and fibrosis parameters**

Pearson correlation between the ten most abundant genera and (A) PELD (B) APRi. (C) total bilirubin and (D) LSM. Pearson correlation between (E ) *Blautia* and PELD at 24 weeks and (F) *Lactobacillus* and LSM at pre-KPE. *The blue line on scatter plots (E-F) indicates the line of best fit determined by linear regression.*

1. Sutton H, Fitzpatrick E, Davenport M, et al. Transient Elastography Measurements of Spleen Stiffness as a Predictor of Clinically Significant Varices in Children. *Journal of Pediatric Gastroenterology and Nutrition* 2018; **67**(4): 446-51.

2. McDiarmid SV, Anand R, Lindblad AS, the Principal I, Institutions of the Studies of Pediatric Liver Transplantation Research G. Development of a pediatric end-stage liver disease score to predict poor outcome in children awaiting liver transplantation1. *Transplantation* 2002; **74**(2).

3. Wai C-T, Greenson JK, Fontana RJ, et al. A simple noninvasive index can predict both significant fibrosis and cirrhosis in patients with chronic hepatitis C. *Hepatology* 2003; **38**(2): 518-26.

4. Kiriyama S, Kozaka K, Takada T, et al. Tokyo Guidelines 2018: diagnostic criteria and severity grading of acute cholangitis (with videos). *Journal of Hepato-Biliary-Pancreatic Sciences* 2018; **25**(1): 17-30.

5. Calinescu AM, Madadi-Sanjani O, Mack C, et al. Cholangitis Definition and Treatment after Kasai Hepatoportoenterostomy for Biliary Atresia: A Delphi Process and International Expert Panel. *Journal of Clinical Medicine*, 2022. (accessed.

6. Zhang J, Kobert K, Flouri T, Stamatakis A. PEAR: a fast and accurate Illumina Paired-End reAd mergeR. *Bioinformatics* 2014; **30**(5): 614-20.

7. Caporaso JG, Kuczynski J, Stombaugh J, et al. QIIME allows analysis of high-throughput community sequencing data. *Nat Methods* 2010; **7**(5): 335-6.

8. Quast C, Pruesse E, Yilmaz P, et al. The SILVA ribosomal RNA gene database project: improved data processing and web-based tools. *Nucleic Acids Res* 2013; **41**(Database issue): D590-6.

9. Huson DH, Beier S, Flade I, et al. MEGAN Community Edition - Interactive Exploration and Analysis of Large-Scale Microbiome Sequencing Data. *PLoS Comput Biol* 2016; **12**(6): e1004957.

10. Love MI, Huber W, Anders S. Moderated estimation of fold change and dispersion for RNA-seq data with DESeq2. *Genome Biol* 2014; **15**(12): 550.
